# Long-Term Exposure to Particulate Matter and Self-Reported Hypertension: A Prospective Analysis in the Nurses’ Health Study

**DOI:** 10.1289/EHP163

**Published:** 2016-05-13

**Authors:** Zhenyu Zhang, Francine Laden, John P. Forman, Jaime E. Hart

**Affiliations:** 1 Department of Epidemiology and Health Statistics, School of Public Health, Zhejiang University, Hangzhou, Zhejiang, China; 2 Channing Division of Network Medicine, Department of Medicine, Brigham and Women’s Hospital and Harvard Medical School, Boston, Massachusetts, USA; 3 Exposure, Epidemiology and Risk Program, Department of Environmental Health, and; 4 Department of Epidemiology, Harvard T.H. Chan School of Public Health, Boston, Massachusetts, USA; 5 Renal Division, Brigham and Women’s Hospital, Boston, Massachusetts, USA

## Abstract

**Background:**

Studies have suggested associations between elevated blood pressure and short-term air pollution exposures, but the evidence is mixed regarding long-term exposures on incidence of hypertension.

**Objectives:**

We examined the association of hypertension incidence with long-term residential exposures to ambient particulate matter (PM) and residential distance to roadway.

**Methods:**

We estimated 24-month and cumulative average exposures to PM10, PM2.5, and PM2.5–10 and residential distance to road for women participating in the prospective nationwide Nurses’ Health Study. Hazard ratios (HRs) and 95% confidence intervals (CIs) were calculated for incident hypertension from 1988 to 2008 using Cox proportional hazards models adjusted for potential confounders. We considered effect modification by age, diet, diabetes, obesity, region, and latitude.

**Results:**

Among 74,880 participants, 36,812 incident cases of hypertension were observed during 960,041 person-years. In multivariable models, 10-μg/m3 increases in 24-month average PM10, PM2.5, and PM2.5–10 were associated with small increases in the incidence of hypertension (HR: 1.02, 95% CI: 1.00, 1.04; HR: 1.04, 95% CI: 1.00, 1.07; and HR: 1.03, 95% CI: 1.00, 1.07, respectively). Associations were stronger among women < 65 years of age (HR: 1.04, 95% CI: 1.01, 1.06; HR: 1.07, 95% CI: 1.02, 1.12; and HR: 1.05, 95% CI: 1.01, 1.09, respectively) and the obese (HR: 1.07, 95% CI: 1.04, 1.12; HR: 1.15, 95% CI: 1.07, 1.23; and HR: 1.13, 95% CI: 1.07, 1.19, respectively), with p-values for interaction < 0.05 for all models except age and PM2.5–10. There was no association with roadway proximity.

**Conclusions:**

Long-term exposure to particulate matter was associated with small increases in risk of incident hypertension, particularly among younger women and the obese.

**Citation:**

Zhang Z, Laden F, Forman JP, Hart JE. 2016. Long-term exposure to particulate matter and self-reported hypertension: a prospective analysis in the Nurses’ Health Study. Environ Health Perspect 124:1414–1420; http://dx.doi.org/10.1289/EHP163

## Introduction

Both short- and long-term exposures to particulate matter (PM) have been shown to be associated with cardiovascular morbidity and mortality in epidemiological studies (Dockery et al. 1993; Hart et al. 2015; Hoek et al. 2013; Laden et al. 2006; Pope et al. 2002; Puett et al. 2008). The mechanisms underlying these associations have been hypothesized to include combinations of autonomic nervous system alterations, systemic inflammation, vascular reactivity, and endothelial dysfunction (Brook et al. 2004, 2010). These mechanisms may also be related to changes in blood pressure and subsequent risk of hypertension. An increasing number of studies have observed that short-term exposures to PM are associated with elevations in systolic and/or diastolic blood pressure and with emergency-department visits for hypertension within several hours to days after air pollution exposure (Arbex et al. 2010; Auchincloss et al. 2008; Chen et al. 2012; Chuang et al. 2010; Dai et al. 2016; Dvonch et al. 2009; Giorgini et al. 2016; Guo et al. 2010; Ibald-Mulli et al. 2001; Mar et al. 2005; Szyszkowicz et al. 2012; Wu et al. 2013). Several studies have also revealed associations between long-term exposures to air pollution and increased blood pressure (Chan et al. 2015; Chuang et al. 2011; Foraster et al. 2014b; Liu et al. 2016; Schwartz et al. 2012).

The evidence for the effects of air pollution on hypertension is inconsistent. Most, but not all, studies from China, Taiwan, and Europe have reported increasing prevalence of hypertension with exposure to particulate matter (PM) and nitrogen oxides (Babisch et al. 2014; Chen et al. 2015; Dong et al. 2013; Foraster et al. 2014a, 2014b; Fuks et al. 2011, 2014; Hoek et al. 2013; Sørensen et al. 2012; Zhao et al. 2013). To date, only two studies have examined the association between exposure to air pollution and incident hypertension. Two analyses have been conducted in the Black Women’s Health Study; positive associations were observed for PM exposures among participants living in Los Angeles, but no associations were observed in the full cohort (Coogan et al. 2012, 2016). Positive associations between exposure to air pollution and hypertension were also observed in an analysis of information from population-based health surveys in Ontario, Canada (Chen et al. 2014).

In the present study, we sought to examine the role of chronic exposures to PM ≤ 2.5 μm in aerodynamic diameter (PM_2.5_), to PM between 2.5 and 10 μm in aerodynamic diameter (PM_2.5–10_), and to PM < 10 μm in aerodynamic diameter (PM_10_), and proximity to major roadways (a proxy for traffic exposure) as risk factors for incident hypertension in the Nurses’ Health Study (NHS), after controlling for a number of time-varying hypertension risk factors. We also explored whether the associations were modified by various lifestyle- and exposure-related factors to determine whether differences in the proportions of susceptible subpopulations may explain the heterogeneity of findings in the literature.

## Methods

### Study Population and Outcome Assessment

The Nurses’ Health Study (NHS) is an ongoing prospective cohort of 121,700 female registered nurses who were between 30 and 50 years of age at the beginning of the study in 1976. The participants originally lived in 11 states (New York, California, Florida, Massachusetts, Pennsylvania, Texas, Ohio, New Jersey, Michigan, Connecticut, and Maryland) but as of the 1990s, at least 10 nurses resided in each state in the contiguous United States. Since the study’s inception, each participant has completed a questionnaire every 2 years, providing information on risk factors, health outcomes, and residential address. From 1976 until the present, only 6% of nurses available for follow-up no longer respond to questionnaires. All nurses who were still living, still responding to questionnaires, and free of hypertension in 1988 (the first year pollution measures were available) were eligible for the present analysis if they had an address in the continental United States where exposure could be assessed. This study was approved by the institutional review board of the Brigham and Women’s Hospital, and informed consent was implied by return of the questionnaires.

On each questionnaire, the women were asked to report any diagnoses they had received since the previous questionnaire. Participants were considered to have hypertension if they reported hypertension on the questionnaire (“physician diagnosis of high blood pressure”). In a validation study (*n* = 100) using medical records to confirm systolic or diastolic BP > 140 or > 90 mmHg, respectively, agreement between the medical record and self-report was nearly 100% (Colditz et al. 1986).

### Exposure Assessment

Geographic information system (GIS)-based spatio-temporal models were used to predict monthly exposures to PM_10_ and PM_2.5_ for each participant residing in the contiguous United States between January 1988 and December 2007. The methods for estimating these exposures have been previously validated and are discussed in detail elsewhere (Yanosky et al. 2014). In brief, we used data from the U.S. Environmental Protection Agency’s (EPA’s) Air Quality System (AQS), the Interagency Monitoring of Protected Visual Environments (IMPROVE) networks, and several Harvard-based research studies, as well as data from various other sources to create separate PM prediction surfaces for each PM size fraction for each month. A GIS was used to generate a number of geospatial predictors including: roadway proximity, percent urban land use within 1 km, smoothed county population density, tract population density, elevation, point sources of PM, and a number of meteorological predictors (Yanosky et al. 2014). Because U.S. EPA AQS monitoring data for PM_2.5_ were not available before 1999, separate PM_2.5_ models were developed for pre-1999 and post-1999 periods (Yanosky et al. 2014). We modeled PM_2.5_ in the period before 1999 using data for PM_10_. We also obtained data on PM_2.5–10_ by subtracting the monthly PM_2.5_ prediction from the monthly PM_10_ prediction at each location. Cross-validation results showed that there was little bias and a high degree of precision when comparing the predicted and observed values (Yanosky et al. 2014). We averaged the monthly-specific exposures to PM_10_, PM_2.5_, and PM_2.5–10_ to create two time-varying exposure metrics; a 24-month moving average and a cumulative average including all predictions from 1988 through the current time period. The 24-month average was chosen to match the reporting periods for hypertension and was calculated using the same 24 months for all nurses in each biennial cycle.

Roadway proximity was used as a proxy for traffic-related air pollution exposures. We calculated distance to roads (in meters) for each residential address using GIS (ArcGIS, version 9.2; ESRI). ESRI StreetMap Pro 2007 road segments were selected to include the three largest U.S. Census Feature Class Codes: A1 (primary roads, typically interstate highways, with limited access, division between the opposing directions of traffic, and defined exits), A2 (primary major, noninterstate highways and major roads without access restrictions), or A3 (smaller, secondary roads, usually with more than two lanes). According to the distribution of roadway proximity in this cohort and previous studies showing exponential decay in exposures with increasing distance, we created the following categories for all road segments combined (A1–A3): 0–99 m, 100–199 m, and > 200 m (Adar and Kaufman 2007; Hart et al. 2009; Karner et al. 2010; Lipfert and Wyzga 2008; Puett et al. 2009). We also examined these distance categories for each type of road segment separately, for the two largest road types (A1–A2), and we considered continuous measures of exposure. To determine the robustness of our findings to cut-point selection, we examined additional categorizations (e.g., 0–49 m, 50–99 m, 100–199 m, 200–499 m, > 500 m; 0–49 m, 50–99 m, 100–199 m, 200–499 m, 500–999 m, > 1,000 m).

### Potential Confounders and Effect Modifiers

Information on potential confounders and effect modifiers was available from each biennial questionnaire (every other questionnaire for dietary information and physical activity) and was modeled as time-varying (with the exception of race). We selected *a priori* variables that have previously been associated with hypertension or with exposure to PM in this cohort as potential confounders: race, physical activity in metabolic equivalent hours per week (MET hours/week), alcohol consumption (grams/day), smoking status (current, former, never) and pack-years, body mass index (BMI; kilograms per meter squared), family history of hypertension, physician-diagnosed diabetes, hypercholesterolemia, menopausal status, nonnarcotic analgesic intake [nonsteroidal antiinflammatory drugs (NSAIDs), acetaminophen, aspirin], and current use of statins. The Dietary Approaches to Stop Hypertension (DASH) score (Bhupathiraju and Tucker 2011) was calculated from each of the semiquantitative food frequency questionnaires. Census-tract median household value and median family income were considered as measures of area-level socioeconomic status (SES). Individual-level SES measures included educational attainment, marital status and partner’s educational attainment, and occupation of the nurse’s mother and father. There is some epidemiologic evidence of increasing blood pressure with increasing latitude, which is hypothesized to be a result of reduced ultraviolet (UV) exposure, colder weather, or differences in flora and fauna (He et al. 1995; Rostand 1997). Therefore, in addition to region of residence (Northeast, South, Midwest, and West), we also controlled for latitude (0°–20°, 20°–40°, 40°–60°) to adjust for potential regional differences in pollution sources and diagnostic patterns. To assess the impact of each potential confounder, we added each variable or set of variables to the basic model including age, race, calendar year, and region of residence. Variables that are known risk factors for hypertension and those that led to a ≥ 10% change in the main effect estimate were included in the final multivariable models. Effect modification by age, diabetes, obesity (BMI ≥ 30 kg/m^2^), DASH score, region, latitude, time period (dichotomized at the year 2000), and whether the participant had moved in the last questionnaire cycle was evaluated through stratification, and statistical significance was assessed using multiplicative interaction terms.

### Statistical Analysis

Time-varying Cox proportional hazards models on a biennial time scale, stratified by age in months and 2-year calendar period (to tightly adjust for trends over time), were used to model the relationship of incidence of hypertension to the predicted PM_2.5_, PM_10_, and PM_2.5–10_ exposure measures. We calculated hazard ratios (HRs) and 95% confidence intervals (CIs) for a 10-μg/m^3^ increase in each size fraction separately and, after examining the correlations between size fractions, in models including both PM_2.5–10_ and PM_2.5_. We also assessed associations of hypertension with roadway proximity using continuous and categorical variables. The linearity of all continuous exposure–response functions was assessed using cubic regression splines. Person-months of follow-up time were calculated from baseline (30 June 1988) until self-reported hypertension, censoring (loss to follow-up, moving outside the contiguous United States), death, or end of follow-up (30 June 2008), whichever came first.

## Results

During the full period of follow-up, the mean age of the participants was ~61 years, most of the participants were never (44%) or former smokers (41%), and 56% had a BMI < 25 kg/m^2^ (Table 1). Mean [± standard deviation (SD)] levels of PM_10_, PM_2.5_ and PM_2.5–10_ exposures in the previous 24 months were 22.24 ± 6.64, 15.61 ± 4.24, and 10.56 ± 4.80 μg/m^3^, respectively. The correlations between exposures are shown in Table 2. Overall, the two exposure averaging periods were highly correlated for each of the size fractions of PM. The correlations between exposures varied: PM_10_ and PM_2.5–10_ were highly correlated, but PM_2.5–10_ and PM_2.5_ were not.

**Table 1:** Age-standardized characteristics of 74,880 participants in the Nurses’ Health Study throughout follow-up (1988–2008).

Characteristic	Mean (SD) or %
Age, years^*a*^	60.39 (8.62)
24-month average PM_10_ (μg/m^3^)	22.24 (6.64)
24-month average PM_2.5–10_ (μg/m^3^)	10.56 (4.80)
24-month average PM_2.5_ (μg/m^3^)	15.61 (4.24)
Body mass index (kg/m^2^)	25.27 (4.54)
Census-tract median income (USD)	65,401 (25,730)
Census-tract median home value (USD)	177,303 (133,832)
Race
White	95
Black	1
Asian	1
Other	4
Body mass index (kg/m^2^)
< 18	3
18–25	53
25–30	31
> 30	13
Alcohol consumption (g/day)
0	31
1–4	26
5–9	9
10–14	7
15–29	5
> 30	3
Missing	18
DASH diet score
Quintile 1	17
Quintile 2	16
Quintile 3	18
Quintile 4	15
Quintile 5	16
Missing	18
Smoking status
Current	14
Former	41
Never	44
Physical activity (MET hr/week)
Quintile 1	15
Quintile 2	17
Quintile 3	18
Quintile 4	19
Quintile 5	19
Missing	12
Family history of hypertension	37
Diabetes	3
Hypercholesterolemia	33
Current statin use	5
Current aspirin use (days/week)
< 1	47
1	16
2–3	7
4–5	4
> 6	11
Individual-level socioeconomic status	
RN degree	81
Married	72
Husband’s education
Less than high school	4
High school	28
More than high school	42
Mother’s occupation
Homemaker	64
Job outside of home	36
Father’s occupation
Professional	27
Other type of job	73
Latitude (degrees)
0–35	18
35–40	18
40–60	65
Region of residence
Northeast	52
Midwest	18
West	14
South	16
Abbreviations: DASH, Dietary Approaches to Stop Hypertension; MET, metabolic equivalent; PM_2.5_, particulate matter ≤ 2.5 μm in aerodynamic diameter; PM_2.5–10_, particulate matter between 2.5 μm and 10 μm in aerodynamic diameter; PM_10_, particulate matter < 10 μm in aerodynamic diameter; SD, standard deviation. Values are means (SD) or percentages and are standardized to the age distribution of the study population. ^***a***^Value is not age adjusted.	

**Table 2:** Spearman correlations between measures of time-varying 24-month and cumulative average particulate matter exposures.

Exposure	24-month average			Cumulative average		
	PM_10_	PM_2.5–10_	PM_2.5_	PM_10_	PM_2.5–10_	PM_2.5_
24-month average
PM_10_	1	0.78	0.66	0.72	0.63	0.56
PM_2.5–10_		1	0.37	0.74	0.88	0.34
PM_2.5_			1	0.73	0.37	0.92
Cumulative average
PM_10_				1	0.81	0.78
PM_2.5–10_					1	0.37
PM_2.5_						1
Abbreviations: PM_2.5_, particulate matter ≤ 2.5 μm in aerodynamic diameter; PM_2.5–10_, particulate matter between 2.5 μm and 10 μm in aerodynamic diameter; PM_10_, particulate matter < 10 μm in aerodynamic diameter.						

There was a total of 960,041 person-years of follow-up and 36,812 incident cases of hypertension among 74,880 women eligible for analysis (incidence rate of 3,834 per 100,000 person-years). HRs and 95% CIs for each 10-μg/m^3^ unit change in 24-month PM and cumulative average predicted PM are presented in Table 3. We present linear exposure–response functions because no statistically significant deviations from linearity were observed. In the basic models, adjusted for age, calendar year, race, and region of the country, each 10-μg/m^3^ increase in PM_10_, PM_2.5_, and PM_2.5–10_ in the previous 24 months was associated with small, but statistically significant, increases in the risk of incident hypertension. The results were similar in models using cumulative average exposures and in multivariable models (24-month average PM_10_ HR: 1.02, 95% CI: 1.00, 1.04; PM_2.5_ HR: 1.04, 95% CI: 1.00, 1.07; and PM_2.5–10_ HR: 1.03, 95% CI: 1.00, 1.07). In models including both PM_2.5_ and PM_2.5–10_, the results were similar to those from the single size-fraction models.

**Table 3:** HRs (95% CIs) of the association of incident hypertension 1988–2008 with each 10-μg/m^3^ increase in particulate matter exposures among 74,880 members of the Nurses’ Health Study.

Exposure	Cases	Person-years	Basic model^*a*^ HR (95% CI)	Multivariable model^*b*^ HR (95% CI)
Single size fraction models
PM_10_
24-month average	36,812	960,041	1.03 (1.01, 1.05)	1.02 (1.00, 1.04)
Cumulative average	36,812	960,041	1.02 (1.00, 1.04)	1.02 (1.00, 1.04)
PM_2.5–10_
24-month average	36,812	960,041	1.04 (1.01, 1.07)	1.03 (1.00, 1.07)
Cumulative average	36,812	960,041	1.04 (1.01, 1.07)	1.03 (1.00, 1.06)
PM_2.5_
24-month average	36,812	960,041	1.05 (1.01, 1.09)	1.04 (1.00, 1.07)
Cumulative average	36,812	960,041	1.02 (0.99, 1.06)	1.01 (0.98, 1.05)
Two size fraction models
24-month average
PM_2.5–10_	36,812	960,041	1.03 (0.99, 1.06)	1.02 (0.99, 1.06)
PM_2.5_	36,812	960,041	1.04 (1.00, 1.08)	1.03 (0.99, 1.07)
Cumulative average
PM_2.5–10_	36,812	960,041	1.03 (1.00, 1.07)	1.03 (1.00, 1.06)
PM_2.5_	36,812	960,041	1.01 (0.98, 1.05)	1.00 (0.97, 1.04)
Abbreviations: CI, confidence interval; HR, hazard ratio; PM_2.5_, particulate matter ≤ 2.5 μm in aerodynamic diameter; PM_2.5–10_, particulate matter between 2.5 μm and 10 μm in aerodynamic diameter; PM_10_, particulate matter < 10 μm in aerodynamic diameter. ^***a***^Adjusted for age, race, calendar year, and region. ^***b***^Additionally adjusted for body mass index (BMI), Dietary Approaches to Stop Hypertension (DASH) diet score, alcohol consumption, smoking status, physical activity, family history of hypertension, menopausal status, nonnarcotic analgesic intake, statin use, diabetes, individual-level socioeconomic status (educational attainment, marital status, partner’s educational attainment, and parental employment), and census-tract median income and home value.				

There was a total of 742,256 person-years of follow-up and 27,906 hypertension cases among the 60,416 women with information on roadway proximity. There was no evidence of elevation in risk of hypertension associated with living close to a major roadway, regardless of the roadway type (Table 4). No associations were observed in continuous models or in models using alternative distance categories. There was no evidence of effect modification by moving status (data not shown).

**Table 4:** HRs (95% CIs) of the association of incident hypertension 1988–2008 with roadway proximity among 60,416 members of the Nurses’ Health Study.

Exposure category	Cases	Person-years	Basic model^*a*^ HR (95% CI)	Multivariable model^*b*^ HR (95% CI)
Distance to A1 (m)
≥ 200	27,173	722,758	1.00 (Referent)	1.00 (Referent)
100–199	509	13,761	0.98 (0.90, 1.07)	0.96 (0.88, 1.05)
0–99	224	5,737	1.02 (0.89, 1.17)	1.01 (0.88, 1.15)
Continuous (per 100 m)	27,906	742,256	1.00 (0.98, 1.01)	1.00 (0.98, 1.01)
Distance to A1–A2 (m)
≥ 200	25,817	688,590	1.00 (Referent)	1.00 (Referent)
100–199	1,244	31,749	1.03 (0.97, 1.09)	1.03 (0.97, 1.09)
0–99	825	21,917	1.01 (0.94, 1.08)	0.97 (0.91, 1.04)
Continuous (per 100 m)	27,906	742,256	0.99 (0.98, 1.00)	1.00 (0.99, 1.01)
Distance to A1–A3 (m)
≥ 200	15,749	423,164	1.00 (Referent)	1.00 (Referent)
100–199	5,495	143,556	1.03 (0.97, 1.09)	1.03 (0.97, 1.09)
0–99	6,662	175,536	1.01 (0.94, 1.08)	0.97 (0.91, 1.04)
Continuous (per 100 m)	27,906	742,256	0.99 (0.98, 1.00)	1.00 (0.99, 1.01)
Abbreviations: A1, primary roads, typically interstate highways, with limited access, division between the opposing directions of traffic, and defined exits; A2, primary major, noninterstate highways and major roads without access restrictions; A3, smaller, secondary roads, usually with more than two lanes; CI, confidence interval; HR, hazard ratio; PM_2.5_, particulate matter ≤ 2.5 μm in aerodynamic diameter; PM_2.5–10_, particulate matter between 2.5 μm and 10 μm in aerodynamic diameter; PM_10_, particulate matter < 10 μm in aerodynamic diameter. ^***a***^Adjusted for age, race, calendar year and region. ^***b***^Additionally adjusted for body mass index (BMI), Dietary Approaches to Stop Hypertension (DASH) diet score, alcohol consumption, smoking status, physical activity, family history of hypertension, menopausal status, nonnarcotic analgesic intake, statin use, diabetes, individual-level socioeconomic status (educational attainment, marital status, partner’s educational attainment, and parental employment), and census-tract median income and home value.				

We did not observe effect modification by diabetes, moving status, region of residence, DASH diet score, or latitude (Table 5; see also Tables S1 and S2). However, the association of PM exposure with hypertension was modified by age and by BMI. *p*-Values for interaction were statistically significant in all models with the exception of age and PM_2.5–10_. Higher risks were observed for younger women (< 65 years old) (HR 24-month average PM_10_: 1.04, 95% CI: 1.01, 1.06; HR PM_2.5_: 1.07, 95% CI: 1.02, 1.12; and HR PM_2.5–10_: 1.05, 95% CI: 1.01, 1.09) and for obese women (BMI ≥ 30 kg/m^2^) (HR 24-month average PM_10_: 1.07, 95% CI: 1.04, 1.12; HR PM_2.5_: 1.15, 95% CI: 1.07, 1.23; and HR PM_2.5–10_: 1.13, 95% CI: 1.07, 1.19). There was a suggestion of higher risks with exposures to PM_2.5_ before 2000.

**Table 5:** HRs for hypertension associated with each 10-μg/m^3^ increase in PM_2.5_ stratified by age, diabetes, obesity, region, DASH score, or latitude.

Effect modifier	Cases	Person-years	24-month average HR^*a*^ (95% CI)	*p*-Value for interaction	Cumulative average HR^*a*^ (95% CI)	*p*-Value for interaction
Current age
< 65	20,823	657,012	1.07 (1.02, 1.12)	0.02	1.05 (1.01, 1.09)	0.008
≥ 65	15,989	303,030	0.99 (0.93, 1.04)		0.96 (0.91, 1.01)
Diabetes
No	35,133	932,718	1.03 (0.99, 1.07)	0.18	1.01 (0.98, 1.04)	0.35
Yes	1,679	27,323	1.14 (0.99, 1.32)		1.08 (0.94, 1.23)
Obesity
No	28,551	822,549	1.01 (0.97, 1.05)	0.0009	1.00 (0.96, 1.03)	0.005
Yes	7,945	124,886	1.15 (1.07, 1.23)		1.10 (1.03, 1.17)
Mover
No	898,002	34,485	1.04 (1.00, 1.08)	0.59	0.90 (0.87, 0.93)	0.89
Yes	62,039	2,327	1.00 (0.88, 1.14)		0.91 (0.82, 1.01)
Time period
1998–2000	22,094	698,971	1.05 (1.01, 1.10)	0.16	0.91 (0.88, 0.95)	0.01
2000–2008	14,718	261,071	1.00 (0.94, 1.06)		0.99 (0.94, 1.05)
Region
Northeast	19,166	503,644	1.01 (0.96, 1.07)	0.53	1.00 (0.95, 1.05)	0.74
Midwest	6,469	168,859	1.07 (0.98, 1.17)		1.06 (0.97, 1.17)
West	4,801	130,349	1.03 (0.97, 1.09)		1.01 (0.97, 1.06)
South	6,360	157,190	1.07 (0.98, 1.17)		1.01 (0.94, 1.09)
DASH
Q1	6,760	166,548	1.07 (0.99, 1.15)	0.57	0.92 (0.86, 0.99)	0.37
Q2	6,125	153,693	1.02 (0.94, 1.10)		0.87 (0.81, 0.94)
Q3	6,854	173,833	1.09 (1.01, 1.18)		0.93 (0.88, 1.00)
Q4	5,780	151,900	1.01 (0.93, 1.09)		0.87 (0.81, 0.93)	
Q5	5,819	159,579	1.05 (0.97, 1.14)		0.88 (0.83, 0.94)
Latitude
Low	6,643	169,691	1.06 (0.99, 1.13)	0.60	1.01 (0.95, 1.07)	0.99
Middle	6,525	168,986	1.03 (0.95, 1.11)		1.01 (0.95, 1.07)
High	23,644	621,365	1.02 (0.97, 1.07)		1.01 (0.96, 1.06)
Abbreviations: CI, confidence interval; DASH, Dietary Approaches to Stop Hypertension; HR, hazard ratio; PM_2.5_, particulate matter ≤ 2.5 μm in aerodynamic diameter. ^***a***^Adjusted for age, race, calendar year and region, body mass index (BMI), DASH diet score, alcohol consumption, smoking status, physical activity, family history of hypertension, menopausal status, nonnarcotic analgesic intake, statin use, diabetes, individual-level socioeconomic status (educational attainment, marital status, partner’s educational attainment, and parental employment), and census-tract median income and home value, as appropriate.						

## Discussion

Long-term exposure to ambient air pollution (PM_10_, PM_2.5_, and PM_2.5–10_) was associated with very small, but statistically significant, increased risks of incident hypertension in this large prospective cohort study of women living throughout the contiguous United States. A 10-μg/m^3^ increase in the 24-month moving average of all three size fractions was associated with the following multivariable adjusted HRs: (PM_10_ HR: 1.02, 95% CI: 1.00, 1.04; PM_2.5_ HR: 1.04, 95% CI: 1.00, 1.07; and PM_2.5–10_HR: 1.03, 95% CI: 1.00, 1.07). Similar results were observed for cumulative average exposures. In addition, associations were stronger among younger women (< 65 years of age) and among the obese; however, there was no evidence of effect modification by diabetes, region or latitude of residence, DASH score, or moving status. Roadway proximity, a proxy for overall traffic exposures, was not associated with incidence of hypertension.

Our estimated HRs for a 10 μg/m^3^ increase in PM_10_. PM_2.5_, and PM_2.5–10_ are lower than the associations that have been observed in some other studies of incident hypertension. In a recent study, 35,303 non-hypertensive Canadian adults responded to population-based health surveys between 1996 and 2005 and were followed until 2010, with a total of 8,649 incident cases of hypertension (Chen et al. 2014). Each 10-μg/m^3^ increase in PM_2.5_ was associated with an HR of 1.11 (95% CI: 1.03, 1.20). A study of 3,236 members of the Black Women’s Health Study (BWHS) living in Los Angeles who were free of hypertension at baseline reported an incident rate ratio (IRR) for hypertension of 1.48 (95% CI: 0.95, 2.31) for each 10-μg/m^3^ increase in PM_2.5_, and the association was attenuated in models that included both PM_2.5_ and NO_x_, with an IRR of 1.32 (95% CI: 0.84, 2.05) (Coogan et al. 2012). However, in analyses based on the full BWHS cohort (*n* = 33,771), an interquartile range increase (2.9 μg/m^3^) in PM_2.5_ was associated with a multivariable adjusted HR = 0.99 (95% CI: 0.93, 1.06) (Coogan et al. 2016). Individuals in the BWHS and Ontario studies tended to be younger than, and a greater proportion were obese compared with, the women in our cohort. However, effect modification by age and BMI was not observed in either of the previous studies (Chen et al. 2014; Coogan et al. 2016).

Several studies have examined the impact of a number of different air pollutants on the prevalence of hypertension, and overall, most have suggested increased prevalence with increasing exposures (Babisch et al. 2014; Chen et al. 2015; Dong et al. 2013, 2015; Foraster et al. 2014a; Fuks et al. 2011; Johnson and Parker 2009; Sørensen et al. 2012). Increases in air pollution have also been associated with increases in the number of emergency-department visits for hypertension in Edmonton, Canada (Szyszkowicz et al. 2012), and with hospital admissions for hypertension in a study from Brazil (Arbex et al. 2010).

There is a large body of literature concentrating on the link between air pollution and blood pressure (Auchincloss et al. 2008; Chuang et al. 2010, 2011; Dai et al. 2016; Fuks et al. 2011; Giorgini et al. 2016; Hoffmann et al. 2012; Kelishadi et al. 2011; Lin and Kuo 2013; Mobasher et al. 2013; Schwartz et al. 2012; Sørensen et al. 2012; Zanobetti et al. 2004). Only a handful of these studies have focused on long-term effects (Auchincloss et al. 2008; Chan et al. 2015; Chuang et al. 2011; Foraster et al. 2014a, 2014b; Fuks et al. 2011; Liu et al. 2016; Schwartz et al. 2012; Sørensen et al. 2012). Overall results have been inconsistent, although the majority of studies have reported positive associations between PM and blood pressure. These inconsistencies may be related to differences in PM composition and to the different targeted study populations [e.g., individuals with prehypertension (Kelishadi et al. 2011), diabetes (Hoffmann et al. 2012), or sleep-disordered breathing (Liu et al. 2016); pregnant women (Mobasher et al. 2013); or participants in cardiac rehabilitation (Zanobetti et al. 2004)].

We found no association of any of our measures of roadway proximity with incident hypertension. The evidence for an association between roadway proximity and hypertension has been mixed in the literature (Dong et al. 2013; Fuks et al. 2014; Johnson and Parker 2009; Kingsley et al. 2015; Kirwa et al. 2014; Sørensen et al. 2012). Studies assessing the association between traffic-related pollutants, such as NO_2_, and hypertension or blood pressure have observed more consistently adverse effects on blood pressure and/or hypertension prevalence (Dong et al. 2013; Foraster et al. 2014a, 2014b; Fuks et al. 2014; Liu et al. 2016; Schwartz et al. 2012; Sørensen et al. 2012; Zhao et al. 2013).

In stratified analyses, we observed stronger effects of air pollution among individuals < 65 years of age; these findings contrasted with those of previous studies that observed no effect modification by age (Chen et al. 2014; Coogan et al. 2016). This observation may reflect a depletion of susceptible individuals in the older age group, or it may reflect true biological differences. Studies have suggested that older individuals exhibit reduced responsiveness to sympathetic and autonomic nervous system stimuli (Cohen et al. 2012; Esler et al. 1995), which could explain the differences in effect by age. It is also possible that differences in time–activity patterns between old and young participants may explain this observation.

We observed a stronger positive association between PM and hypertension in obese participants, similar to two other studies (Dong et al. 2015; Zhao et al. 2013) that reported that obesity may amplify the association of long-term air pollution exposure with hypertension in China. The mechanism underlying the synergistic effects of PM and obesity on hypertension is not clear; one possible explanation is that obesity and exposures to PM both result in systemic inflammation (Dubowsky et al. 2006). Additionally, obese individuals have a higher inhalation rate than their normal-weight counterparts (Brochu et al. 2014). Thus, women with higher BMIs are a potentially susceptible population, and the causal pathway warrants further exploration.

There is a large body of evidence suggesting that PM inhalation leads to the elicitation of systemic inflammation, oxidative stress responses, and endothelial dysfunction, as well as to imbalance of the autonomic nervous system, all of which are plausible mechanisms that may underlie associations with acute and chronic blood pressure elevation (Brook et al. 2010; Donaldson et al. 2001). If there are repeated rises in intravascular pressure, hypertrophic remodeling of the resistance vessels will cause medial thickness, which will result in a fixation of blood pressure elevation (Valavanidis et al. 2008). Components of PM, such as black carbon, have been shown to elevate blood pressure by activation of the sympathetic nervous system, direct vasoconstriction, and alterations in blood coagulability (Schwartz et al. 2012).

This study has a few key limitations. Our findings may not be generalizable to the whole U.S. population because our study participants represent a narrow range of occupation and socioeconomic status, are less obese, and are exposed to lower levels of PM than some of the other populations that have been studied (particularly those in China). Although we used a complex spatiotemporal model to predict address-level monthly exposure estimates, we did not have information on the amount of time each participant spent at home or on the amount of ambient pollution that may have infiltrated the home, which would lead to exposure misclassification. To assess the potential impact of this error, we used measurement error correction methods (Hart et al. 2015; Liao et al. 2011) to estimate the potential impact of using ambient rather than personal estimates of PM_2.5_. In the full cohort, using only follow-up data from after 2000 (a limitation of the method), the HR for a 10-μg/m^3^ increase in PM_2.5_ was 1.07 (95% CI: 1.01, 1.14), and the measurement corrected estimate was 1.12 (95% CI: 1.00, 1.25). This finding suggests that we likely underestimated the effects of PM on hypertension. Another limitation is that because PM_2.5_ and PM_2.5–10_ were estimated from PM_10_ before 1999 (owing to the sparsity of PM_2.5_ monitors), it is likely that there is more measurement error in the exposure estimates in the earlier portions of the study than in the later portions. This measurement error would limit our ability to detect associations; however, in models stratified by time period, we did not observe stronger effects for PM_2.5_ after 2000. Furthermore, roadway proximity is a weak proxy for actual traffic-related exposures, such as gaseous pollutants and noise effects, likely explaining our lack of elevated findings. Additionally, limited person-time in the cohort was spent at addresses within 99 m of A1 to A3 roadways. Another limitation is that although we were able to adjust for a large number of time-varying covariates that were either known risk factors for exposure or were predictors of exposure, the large number of factors included in our multivariable models may have led to over-adjustment. In particular, the inclusion of potential mediators of the air pollution effect may have been problematic. Our outcome measure also may be subject to misclassification, although our population has medical expertise, and they have been shown to provide accurate information on hypertension (Colditz et al. 1986). There may also have been differences in diagnosis patterns that were not fully controlled for, even though our models were adjusted for region of the country and for calendar year. Finally, information on absolute levels of systolic and diastolic blood pressure is not available in the NHS cohort; therefore, a weakness of our study is the reliance on the dichotomous outcome of hypertension.

This study has several strengths, including the availability of monthly estimates of three size fractions of PM at the residential addresses of all cohort members, time-varying data on potential confounders and effect modifiers, and previously validated incidence of hypertension. Additionally, we were able to adjust for various lifestyle factors associated with hypertension, including diet, physical activity, and family history. Most importantly, with the GIS-based exposure model, we could assess exposures on a finer spatial and temporal scale than had been achieved by most previous studies.

## Conclusion

In conclusion, we found small but statistically significant associations of 24-month and cumulative average exposures to PM_10_, PM_2.5_, and PM_2.5–10_ with incidence of hypertension among women in the Nurses’ Health Study living throughout the contiguous United States. The associations were stronger when the analyses were restricted to women < 65 years of age and to the obese. There was no association between incidence of hypertension and residential roadway proximity. Because hypertension is a potential risk factor for cardiovascular disease with a very high prevalence, even small changes are important at the population level.

## Supplemental Material

(252 KB) PDF
