# Supplementary material for: Long-Term Exposure to Particulate Matter and Self-Reported Hypertension: A Prospective Analysis in the Nurses’ Health Study
Source: Environ Health Perspect. 2016 May 13;124(9):1414–20. doi: 10.1289/EHP163 (PMC5010392; doi:10.1289/EHP163)
Supplement: (252 KB) PDF [file EHP163.s001.acco.pdf]

Environ Health Perspect
DOI: 10.1289/EHP163

Note to readers with disabilities: *EHP* strives to ensure that all journal content is accessible to all readers. However, some figures and Supplemental Material published in *EHP* articles may not conform to 508 standards due to the complexity of the information being presented. If you need assistance accessing journal content, please contact ehp508@niehs.nih.gov. Our staff will work with you to assess and meet your accessibility needs within 3 working days.

Supplemental Material

Long-Term Exposure to Particulate Matter and Self-Reported Hypertension: A Prospective Analysis in the Nurses' Health Study

Authors: Zhenyu Zhang, Francine Laden, John P. Forman, and Jaime E. Hart

Table of Contents

Table S1. HRs for Hypertension associated each 10 μg/m^3 increase in PM_{10}, stratified by age, diabetes, obesity, region, DASH score or latitude

Table S2. HRs for Hypertension associated with each 10 μg/m^3 increase in $PM_{2.5-10}$, stratified by age, diabetes, obesity, region, DASH score or latitude

Environ Health Perspect
DOI: 10.1289/EHP163

Table S1. HRs for Hypertension associated each 10 μg/m^3 increase in PM_{10}, stratified by age, diabetes, obesity, region, DASH score or latitude.

Effect Modifier	Cases	Person Years	24-Month Average HR[a] (95%CI)	p-value for interaction	Cumulative Average HR[a] (95%CI)	p-value for interaction
**Current Age**						
< 65	20,823	657,012	1.04 (1.01, 1.06)	0.03	1.03 (1.01, 1.06)	0.009
≥ 65	15,989	303,030	1.00 (0.97, 1.03)		0.99 (0.96, 1.02)	
**Diabetes**						
No	35,133	932,718	1.02 (1.00, 1.04)	0.45	1.02 (1.00, 1.04)	0.87
Yes	1,679	27,323	1.05 (0.97, 1.14)		1.02 (0.95, 1.10)	
**Obesity**						
No	28,551	822,549	1.01 (0.98, 1.03)	0.001	1.01 (0.99, 1.03)	0.004
Yes	7,945	124,886	1.07 (1.04, 1.12)		1.06 (1.03, 1.10)	
**Region**						
Northeast	19,166	503,644	1.01 (0.98, 1.05)	0.76	1.01 (0.98, 1.04)	0.85
Midwest	6,469	168,859	1.04 (0.99, 1.09)		1.03 (0.98, 1.08)	
West	4,801	130,349	1.02 (0.99, 1.05)		1.02 (0.99, 1.05)	
South	6,360	157,190	1.01 (0.94, 1.09)		1.00 (0.94, 1.07)	
**DASH**						
Q1	6,760	166,548	1.04 (0.99, 1.08)	0.87	0.94 (0.91, 0.98)	0.59
Q2	6,125	153,693	1.01 (0.97, 1.06)		0.92 (0.88, 0.95)	
Q3	6,854	173,833	1.01 (0.97, 1.05)		0.93 (0.90, 0.97)	
Q4	5,780	151,900	1.02 (0.97, 1.06)		0.91 (0.88, 0.95)	
Q5	5,819	159,579	1.03 (0.99, 1.07)		0.91 (0.88, 0.94)	
**Latitude**						
Low	6,643	169,691	1.04 (1.00, 1.08)	0.46	1.03 (1.00, 1.07)	0.50
Middle	6,525	168,986	1.02 (0.98, 1.07)		1.02 (0.98, 1.06)	
High	23,644	621,365	1.01 (0.98, 1.04)		1.01 (0.98, 1.03)	

[a] Adjusted for age, race, calendar year, region, BMI, DASH diet score, alcohol consumption, smoking status, physical activity, family history of hypertension, menopausal status, non-narcotic analgesic intake, statin use, diabetes, individual level socioeconomic status (educational attainment, marital status, partner's educational attainment, and parental employment), and Census tract median income and home value, as appropriate

DOI: 10.1289/EHP163

Table S2. HRs for Hypertension associated with each 10 µg/m^3 increase in $PM_{2.5\text{-}10}$, stratified by age, diabetes, obesity, region, DASH score or latitude.

Effect Modifier		Cases	Person-Years	24-Month Average HR[a] (95%CI)	p-value for interaction	Cumulative Average HR[a] (95%CI)	p-value for interaction
**Current Age**							
	< 65	20,823	657,012	1.05 (1.01, 1.09)	0.11	1.04 (1.01, 1.08)	0.13
	≥ 65	15,989	303,030	1.00 (0.96, 1.05)		1.01 (0.97, 1.05)	
**Diabetes**							
	No	35,133	932,718	1.03 (0.99, 1.06)	0.76	1.03 (1.00, 1.06)	0.85
	Yes	1,679	27,323	1.05 (0.93, 1.17)		1.02 (0.91, 1.14)	
**Obesity**							
	No	28,551	822,549	1.00 (0.97, 1.04)	<0.0001	1.01 (0.98, 1.05)	0.006
	Yes	7,945	124,886	1.13 (1.07, 1.19)		1.09 (1.04, 1.15)	
**Region**							
	Northeast	19,166	503,644	1.04 (0.97, 1.10)	0.36	1.03 (0.97, 1.08)	0.83
	Midwest	6,469	168,859	1.08 (1.00, 1.16)		1.04 (0.97, 1.12)	
	West	4,801	130,349	1.03 (0.98, 1.07)		1.04 (0.99, 1.08)	
	South	6,360	157,190	0.98 (0.90, 1.06)		0.99 (0.91, 1.08)	
**DASH**							
	Q1	6,760	166,548	1.07 (1.00, 1.13)	0.48	0.95 (0.90, 1.01)	0.69
	Q2	6,125	153,693	1.03 (0.97, 1.10)		0.93 (0.87, 0.98)	
	Q3	6,854	173,833	0.99 (0.93, 1.05)		0.91 (0.86, 0.96)	
	Q4	5,780	151,900	1.03 (0.96, 1.09)		0.91 (0.86, 0.97)	
	Q5	5,819	159,579	1.02 (0.96, 1.08)		0.90 (0.85, 0.96)	
**Latitude**							
	Low	6,643	169,691	1.05 (1.00, 1.11)	0.88	1.06 (1.01, 1.11)	0.43
	Middle	6,525	168,986	1.03 (0.98, 1.10)		1.04 (0.98, 1.10)	
	High	23,644	621,365	1.04 (0.99, 1.10)		1.02 (0.97, 1.06)	

[a] Adjusted for age, race, calendar year, region, BMI, DASH diet score, alcohol consumption, smoking status, physical activity, family history of hypertension, menopausal status, non-narcotic analgesic intake, statin use, diabetes, individual level socioeconomic status (educational attainment, marital status, partner's educational attainment, and parental employment), and Census tract median income and home value, as appropriate
